# A narrative inquiry into the agency of an EFL teacher in Western China

**DOI:** 10.3389/fpsyg.2022.957372

**Published:** 2022-09-21

**Authors:** Haibo Gu, Yulian Liang, Qian Wang

**Affiliations:** ^1^School of Foreign Languages, Soochow University, Suzhou, China; ^2^School of Foreign Languages, Xinjiang Normal University, Ürümqi, China; ^3^Wuxi Foreign Language School, Wuxi, China; ^4^Academy of Future Education, Xi’an Jiaotong-Liverpool University, Suzhou, China

**Keywords:** narrative inquiry, teacher agency, language teacher, Western China, under-resourced environment

## Abstract

Despite the increasing interest in teacher agency in the field of language teacher psychology, little attention has been paid to how language teachers enact their agency in an under-resourced environment. To address the research gap, this narrative study explored how a secondary English as a foreign language teacher in Western China enacted his agency for professional development and identified its sources. The findings revealed that this teacher enacted his agency through passionate exploration of adaptive teaching and continuous investment in autonomous learning. His agency was attributed to the interplay of his past experiences, long-term goals, teaching beliefs, and the challenging working environment. Consequently, implications for teacher agency research and practice are discussed.

## Introduction

Teacher agency has become an important research focus, attracting growing interest in the field of language education (Tao and Gao, 2017; Bao et al., 2020; Huang and Yip, 2021). The development of agency is influenced by individuals’ characteristics and relationships with their surrounding contexts, which determine whether agentic choices can be put into practice (Jiang and Zhang, 2019; Bonner et al., 2020). Teachers with agency tend to perceive themselves as active learners who can act decisively and reflect thoroughly on the impact of their actions (Priestley et al., 2015). Moreover, when teachers are confident about practicing agency, they are more likely to consider their job as a meaningful profession (Priestley et al., 2015). Such a belief in turn strengthens their commitment to being effective teachers and generates additional efforts toward their professional development. Therefore, teacher agency plays a key role in promoting the professional practice of language teachers (Bao et al., 2020). More concretely, teacher agency can be observed in attempts to build rapport with students, resist normative discourses in the school, reflect on teaching methodology, and initiate positive changes in student learning that align with their educational beliefs (Roberts and Graham, 2008).

Many studies of language teacher agency have been carried out in the context of curriculum reform (e.g., Priestley et al., 2015; Tao and Gao, 2017; Wei and Chen, 2019; Ruan et al., 2020). However, as due to COVID-19, different types of anxiety emerged among high school EFL teachers with Livestream teaching (Liu et al., 2022), a series of studies has been conducted to examine the agency of teachers (Chaaban et al., 2021; Damşa et al., 2021; Ashton, 2022), but only a few studies have discussed teacher agency in under-resourced environments (e.g., Ebersöhn and Loots, 2017; Gaddefors et al., 2020). Teachers who feel creative and deliberate in unproblematic situations may not achieve high levels of agency, as they simply follow the norm (Priestley et al., 2015). Conversely, problematic contexts like an under-resourced environment may play a significant role in stimulating teachers to enact agency in their teaching (Toom et al., 2015). Therefore, it is necessary to examine the agency of teachers in under-resourced environments. However, the lack of empirical research on EFL teachers’ agency in such environments creates ambiguity when trying to understand how EFL teachers enact their agency in a significantly more complex and challenging context. Thus, little can be done to help such EFL teachers improve their teaching and professional development.

It has been pointed out that the achievement of agency results from the interplay of individual efforts, available resources, and contextual and structural factors as they converge in unique situations (Biesta and Tedder, 2007). In particular, EFL teachers in an under-resourced environment rely more on personal agency to promote the development of English teaching. Thus, to create a positive language teaching and learning environment, efforts should be made to understand EFL teachers’ agency in under-resourced teaching contexts to facilitate their professional development.

With a deep concern regarding the aforementioned issue, this narrative research aims to explore the agency of an EFL teacher in Western China by collecting data through semi-structured interviews and case documents. Accordingly, it is hoped that a deeper understanding of the sources of an EFL teacher’s agency could generate implications for other EFL teachers in similar contexts to improve their teaching and learning.

## Literature review

Informed by different theoretical perspectives, scholars have conceptualized teacher agency as an intentional act, a sociocultural mediated capacity, a phenomenon, and a discursive practice (Tao and Gao, 2021). Several factors could impact the ways and the extent to which teachers exercise agency. Rather than seeing agency as a capacity held within individuals, the ecological view of agency considers it as “an emergent phenomenon of the ecological conditions through which it is enacted” (Biesta and Tedder, 2007, p. 137). First, it emphasizes the complexity involved when teachers form a sense of agency (Teng, 2019) and highlights teachers’ abilities to utilize their environments rather than simply being in their environments. According to the ecological perspective, the achievement of agency will always result from the interplay of individual efforts, available resources, and contextual and structural factors (Biesta and Tedder, 2007).

Second, the ecological view also posits that time plays an essential role in developing agency. As Ashton (2022) states: “enacting agency in practice within an ecological perspective involves a process of teachers reflecting on their past experiences, setting future goals and plans, and evaluating these to identify appropriate courses of action” (p. 2). According to Priestley et al. (2015), teacher agency should be understood “as a configuration of influences from the past (i.e., the experience and expertise teachers bring to their work), orientations toward the future (i.e., the orientations that guide teachers’ work), and engagement with the present (the decisions that teachers make on what to do and how to do it in the present state)” (p. 33).

Furthermore, Priestley et al. (2015) developed a model to better understand teacher agency. It outlines three dimensions that support the development of agency: iterational, practical-evaluative, and projective. This model suggests that the achievement of agency is informed by both professional and personal past experiences (iterational); it acknowledges that the achievement of agency is oriented toward the future in some combination of short(er)-term and long(er)-term objectives, values, and aspirations (projective); and it stresses that “agency is always enacted in a concrete situation—both constrained and supported by discursive, material, and relational resources available to actors” (p. 40; practical-evaluative). This study posits that the ecological approach provides a more comprehensive understanding of teacher agency; therefore, it is the conceptual framework chosen for this study.

There has been an increasing tendency among scholars to focus on teachers’ agency in different contexts (Priestley et al., 2015; Kayi-Aydar et al., 2019; Tao and Gao, 2021). Recently, Ashton (2022) examined the agency of language teachers by studying their response to emergency online teaching in the unprecedented educational situation of the COVID-19 pandemic. By discussing the unique critical incidents that prompted teachers to enact agency and the factors influencing their actions, it provides additional evidence on how social structural factors influence teacher agency. Likewise, Damşa et al. (2021) explored the nature and degree of university teachers’ agency and emergency responses in Norway in the first month of the lockdown due to the COVID-19 crisis. This qualitative study unveiled different scenarios of enacting teachers’ agency, both ostensible and occlusive, whereby actions were shaped by the constrained circumstances.

A growing body of empirical studies has examined teacher agency in education reform. For example, Tao and Gao (2017) examined how Chinese teachers enact agency to facilitate their professional development during curricular reform in a Chinese university. They find that teachers’ learning, teaching, and research endeavors concerning the new curriculum are directed by various identity commitments and enacted in highly individualized ways mediated by their prior experiences. Similarly, Ruan et al. (2020) conducted a thematic analysis of data from semi-structured interviews, classroom observations, and journal entries to explore how English teachers in a Chinese university exercised their agency in a climate of reforms when self-discrepancies emerged in their classroom instruction, showing that teachers tended to exercise their agency in various meaning-making efforts to enhance their teaching effectiveness in the classroom in line with their prior experiences, professional knowledge, and actual selves.

Moreover, some studies have situated the research context in the basic education environment. For instance, Wei and Chen (2019) explored teacher agency in junior high schools with advanced teaching materials and resources. In this study, a sociocultural perspective was adopted to understand two new science teachers’ manifestation of agency and the interaction between their identity and agency. They pointed out that the two participants’ agency contrasted in terms of perspectives and actions, which manifested in six spaces: (1) personal characteristics, (2) personal beliefs, (3) interactions with students, (4) interactions with colleagues, (5) curriculum materials, and (6) high-stakes examinations. Findings demonstrate that the two participants’ contrary actions were the results of different perspectives and beliefs.

Furthermore, in a recent study by Huang and Yip (2021), a triadic reciprocity framework was adopted to analyze how three Hong Kong secondary English-as-a-second-language (ESL) teachers exercised their teacher agency to take control of their teaching and professional development. Their findings reveal that these teachers exercised different degrees of proactive, reactive, and passive agency, which were, in turn, influenced by four properties of human agency, i.e., intentionality, forethought, self-reactiveness, and self-reflectiveness, when they responded to personal, behavioral, and environmental determinants.

In addition, very few teacher agency studies have focused on under-resourced teaching contexts. A case in point is Pei and Yang (2019), who conducted a large-scale 4-year-project focused on improving the learning and education quality for ethnic minority students through a teacher training program in Yunnan Province that investigated how professional teacher development training activates rural teacher’s agency in transforming their practice and becoming self-regulated practitioners in China. In this case study, the teachers’ agency shifted from initial resistance to adopting the training, and finally to innovation in practice, during which both their cognition and practices were transformed.

The studies presented above indicate that teachers’ professional agency varies in its manifestations across individuals and situations and under different levels according to various aspects of a teacher’s professional work (Huang and Yip, 2021). While most research has contributed to our understanding of teachers’ agency in different situations, there remain limited studies focused on the manifestations and sources of teacher agency in under-resourced teaching contexts. To address this gap, this narrative study aims to deepen our understanding of teacher agency exercised by an EFL teacher in an under-resourced teaching environment in Western China. More specifically, the study addresses the following two research questions:

(1) How does the EFL teacher enact his agency in teaching and learning?
(2) What are the sources of the EFL teacher’s agency in the under-resourced environment?

## Methodology

Through narrative inquiry (Clandinin and Connelly, 2000), the present study explored how an EFL teacher, Louis, actively enacted his agency in both teaching and learning in Western China. Narrative inquiry is an effective means of “getting at what teachers know, what they do with what they know, and the sociocultural contexts within which they teach and learn to teach” (Golombek and Johnson, 2004, p. 308). Narrative research has become an important means for understanding teachers as knowers of their situations, of teaching, and of learning (Clandinin and Connelly, 1998). Furthermore, Connelly and Clandinin (1987, p. 134) noted that “Narrative is concerned with specific, concrete events in a person’s life and is concerned to give an account of a person, offering an interpretive reconstruction of parts of a person’s life.” In light of this perspective, in this study, this method was applied to deeply understand the participant’s agency stories and agency sources. The study of teachers’ narratives—that is, stories of teachers’ own experiences—is increasingly seen as crucial to the study of teachers’ thinking, culture, and behavior (Connelly and Clandinin, 1987).

### The participant and context

The participant of this study was a 38-year-old male middle school English teacher named Louis, who was selected based on two criteria. First, a high level of agency was reflected in his words and performance during his participation in a teacher training program at a university where the researchers met him. In the training course, Louis was found to be the most active learner, eager, and keen to participate in interactions with the first researcher of this study. Second, when we invited teachers to engage in extended conversation at night, Louis was the only one who came to the office, which provided further evidence of his agency. More importantly, he was willing to share his stories and feelings, which allowed data collection to be conducted smoothly.

To better understand the research context, a brief introduction to the teacher’s background information is provided, particularly his learning and teaching experiences (see Table 1). Born and raised in a family with regular access to newspapers and books, Louis was keen on reading and learning. However, living in remote area far from a major city, Louis had little contact with English, so he could learn nothing except what could be found in textbooks. Shortly after participating in a Canadian summer camp in high school, his interest in learning English was kindled. Afterward, his curiosity prompted him to search for and create different learning resources and environments in his own life. From his past positive learning experiences with English learning, Louis’ determination to inspire more students in this area to learn English grew. After graduating from T University in 2007, he attended an English teacher training for 1 month at L Normal School, after which he was employed as a public school English teacher in a remote rural area. Constrained by the geographical and social economic environment, education development in this area is both challenging and complex. However, when he witnessed significant changes due to the new education policy, Louis began to pay more attention to maximizing students’ potential for learning English and embarked on his journey toward establishing a solid teaching practice.

**TABLE 1:** Louis’s profile.

Year	Activities
1994–1999	Primary school
1999–2000	Middle school: Had no regular English classes or textbook
2001–2003	High school: Met a Canadian at summer camp
2003–2007	Majored in Tourism Management from T University
2007–2010	1-month training in L Normal University; 3°years teaching in a nomadic school
2010-now	Teaching in T Middle School
2014	Joined a teacher training program (Total Immersion Program)
2015–2019	Taught phonics for 4°years; set up an English corner in school
2015–2020	Managed to find some teaching materials about phonics; recompiled a phonics textbook
2021	Initiated a teaching program for 30 teachers (both primary and middle school)

### Data collection

Aiming to understand the complexity of teacher agency in this study, the primary source of data was in-depth interviews with the participant. Two interviews with Louis were conducted, spanning two semesters for the academic year from 2020 to 2021 in October 2020 and July 2021. The first interview, which lasted 1 h and 40 min, was conducted to understand the participant’s background information and explore his practices in teaching and learning as an EFL teacher in Western China. The second interview, which lasted 2 h, allowed the participant to reflect on his teaching practices in the past 6 months and the changes that had occurred. The two interviews overall lasted 3 h and 40 min, and both were audio-recorded with the participant’s permission. The two interviews were semi-structured, framed around several general topics that allowed the interviewee to freely talk about his own experiences and feelings and the interviewer to delve deeper into the interviewee’s accounts. After completing the interviews, a summary was made. The interviews were conducted with the participant in English to ensure that he could express himself freely. The researcher did not stick to the interview outline when asking questions, so as to allow the topic to be appropriately adapted and to elicit more critical stories from the participant according to the actual progress of the interview (Seidman, 2013).

Besides formal interviews, the researchers also engaged in personal communication with Louis through WeChat, one of the most popular social communication apps used in China. While this type of data was not subjected to systematic analysis, it generated insights for the understanding and interpretation of the participant’s agency. Notably, this form of informal interaction helped the researcher establish rapport with Louis so that he could comfortably share stories and feelings that were personally meaningful and significant.

Case documents also play a vitally important role in proving the trustworthiness and authenticity of the interview data, providing supplementary information on the participant. The case documents of this study involve the participant’s revised story fragments, learning materials he compiled for students, chat records with the researcher, and some screen-shots of his social media moments and shared teaching videos.

### Data analysis

The interviews were transcribed, after which the transcripts were sent to the participant for accuracy check. The participant gave timely feedback on the transcribed interviews.

To answer the first research question, narrative analysis, which aims to construct stories from the available information, was used to analyze the data. In this phase, stories were written about how the participant enacted his agency. Each story was based on in-depth interviews exploring the EFL teacher’s teaching and learning experiences. After reading the interview data thoroughly, plots were developed that fit the data and are characterized by an exposition, complication, climax, falling action, and resolution (Gorden and Kuehner, 1999). The exposition consisted of a description of the background in which the story took place, and the complication contained a description of the participant’s practices. The climax involved the problems and conflicts the EFL teacher encountered, and the falling action concerned the changes he made or actions he took after the conflicts. The resolution included the results and outcomes. Moving back and forth between the data and the plot clarified which aspects, situations, and contexts contributed to the teacher’s agency (Leeferink et al., 2015). This process generated two stories about how Louis enacts his agency in teaching and learning, including “Passionate Exploration of Adaptive Teaching” and “Continuous Investment in Autonomous Learning.”

To answer the second research question, qualitative content analysis was conducted. Qualitative content analysis refers to “a systematic method for searching out and describing meaning within texts” (Drisko and Maschi, 2016, p. 87), and focuses on identifying categories or themes that both summarize the content found in the full data set and highlight the key content. In this study, it included two levels: open coding and selective coding (Strauss and Corbin, 1998). During the open coding stage, the transcripts were read repeatedly while noting initial impressions and searching for various meanings. Throughout the process, concise notes were taken of the events the participant described as significant and the meanings associated with the events. In addition, the researcher’s thoughts and impressions were used as cues for the following data analysis phase. During the selective coding stage, a top-down approach was implemented. The researcher developed an initial coding scheme based on the conceptual framework adapted from the research model and pilot coding of the two interviews. In total, three categories of the sources of the agency (practical-evaluative, iterational, and projective) were coded. Each category had sub-categories. Taking “I have been paying more attention to researching how to maximize my students’ learning potential in English” as an example, it is coded into “striving for students’ future development,” a subcategory of “long-term goals.” Another graduate student was invited to simultaneously code the same interview transcripts with the initial coding scheme. Then, after discussing differences and reconfirming appropriate codes with fellows, a modified coding scheme was obtained.

## Findings

Based on data analysis, this study concluded two stories that depict the agency of this English teacher. The following narrate two stories about how the EFL teacher in Western China enacted his agency in both teaching and learning and then elaborate on the sources of the teacher’s agency.

### The EFL teacher’s agency stories

#### Passionate exploration of adaptive teaching

Louis, a middle school English teacher, had 15 years of teaching experience in which students’ test scores were increasingly emphasized. However, Louis did not believe that tests were paramount in education. He stressed, “tests are not good for students’ future. They fail to test students’ language proficiency. Although some students performed well in tests, they could not speak English” (Interview 1, 28 Nov. 2020). Instead, his major teaching goal was to improve students’ speaking skills and foster students’ interest in lifelong English learning. This idea of teaching was partly derived from his past learning experiences. As he recalled:

*During middle school, I ^1^ did not have any English learning experience because nobody taught me. Since I lived within the boundary of this area, I had never heard any English words. The textbook was old, and teachers only taught those words in it. (Interview 1, 28 Nov. 2020*)

In 2014, influenced by his friend Josh, Louis came into contact with TIP.^2^ Since then, he has developed the idea of practicing the Phonics Teaching Method^3^ in his teaching, which he thought could be beneficial for his students to improve English speaking. Hence, Louis set up an English corner in his school for teaching students phonics every weekend, which he ran for 4 years:

*I taught them for free and found it was beneficial. My efforts seemed to pay off. The students in the English corner made rapid progress in English speaking. Even if one student can speak as well as I do, I am pretty sure that my method is right. If it means there are some possibilities, I will continue doing it. (Interview 1, 28 Nov. 2020*)

Unlike other EFL teachers in his school, Louis was not satisfied with teaching to the test or conducting a lesson aimed at making students obedient. Accordingly, he strove to build a supportive English learning environment for students by engaging them in various learning activities with authentic language input. Once, when going to S City to attend a teacher training program, Louis observed a class being taught in X Middle School. To his surprise, the students there could speak English quite fluently, displaying completely different language proficiency from students in his school. Shocked by their performance, Louis exclaimed:

*I want to see that happen in the future. I should make some changes for all the students in our school. Let them speak English like this. I think it just takes time. (Interview 2, 7 July 2021*)

Inspired by the teacher training experiences, Louis tried classroom activities such as group discussions and pair works in his classes, setting situations to promote English conversation among students. Furthermore, he brought innovative activities, including brainstorming and drawing mind-maps to his class, aiming to cultivate students’ imagination and creativity. Regarding this, he noted, “We should collect various teaching materials that someday we can use in our class. It is good for students and our teaching” (Interview 2, 7 July 2021). As an example, after reading some of Shakespeare’s plays himself, Louis made some adaptations of the lines for students to read. He also introduced videos with short conversations for students to imitate.

However, when putting these methods into practice, Louis was disappointed to find that his students’ English proficiency was very low, which meant many activities could not be carried out smoothly. What is worse, when checking students’ learning after class or the following day, Louis noticed that they seemed to have learned nothing, which depressed him. In addition, Louis expressed his concern that English was treated as an unimportant discipline in his context:

*Most primary school teachers pay little attention to teaching English because its proportion in the “xiaokao” had been changed into 15 scores (all the subjects add up to 400 scores). Only a few schools have English classes once or twice a week. All English teachers are required to teach other subjects, so students’ English proficiency is much lower than before. (Interview 2, 7 July 2021*)

Given the reflections on the reasons for the low English proficiency of students, Louis began creating opportunities to train primary school teachers on how to teach phonics, thus motivating them to improve their language proficiency. In 2021, he organized an online learning community with approximately 30 primary school teachers to facilitate learning English online with support from the school’s educational policy:

*I think the problem with students’ English proficiency is the problem of the teachers’ English proficiency, so I want to help teachers become better. If they are better, they will talk about English with their students. Thus, the students can receive a good English education. (Interview 2, 7 July 2021*)

Likewise, families in this area are not overly concerned about what their children learn at school but only whether they are healthy. This comes down to the fact that people in this environment generally believe that education is a matter of school, and they lack sufficient understanding of the educational function of the family. Realizing this issue, Louis realized that he must shoulder the responsibility of educating these children more. This value guided him to integrate extra knowledge and content into his teaching content:

*I help them learn about high technology. For example, I let them see augmented reality, virtual reality, and 3D images. Sometimes I also let them watch videos about artificial intelligence since I hope they can open up their mind. That is very important. (Interview 1, 28 Nov. 2020*)

It seemed that while Louis put significantly more effort into teaching than other teachers, the school used students’ test scores to evaluate teachers’ performance, and the students’ grades in his class continuously lagged behind those of other classes. As a result, he was often reminded by the schoolmaster, “We need the score. The score is the truth” (Interview 1, 28 Nov. 2020). At home, his wife blamed him as well, “You poured much passion into your students, but I did not see any result. You should learn from the women teachers on how to teach students” (Interview 1, 28 Nov. 2020). Louis described the examination system as a shackle constraining his teaching practice that restricts EFL teachers to teaching the students only what they will be tested on. In Louis’ words, “The parents and teachers only want to see a good result in a short period. They never see how the students will unconsciously be influenced in their future” (Interview 1, 28 Nov. 2020). However, considering students’ graduation, Louis expressed his concerns about students’ grades as well. Accordingly, he had made some adjustments in his teaching practice, such as dividing the class into different levels and assigning different learning tasks.

In summary, Louis developed a strong sense of agency in his teaching process as he took agentic actions to adapt his teaching, thus promoting students’ learning. His belief in educational values and his desire to teach educationally rather than instrumentally are significant projective agency sources for him to negotiate with the test-oriented culture (Biesta et al., 2015).

#### Continuous investment in autonomous learning

Living far away from the city, Louis said he could barely hear anyone speak English during his school days. Louis confessed that his interest in English was sparked for the first time when he had the opportunity to have authentic communication with foreigners from a Canadian summer camp. Since then, driven by a great interest in learning English, Louis has been taking a tortuous path on the road of learning English through ongoing investment in searching for available learning resources and building learning communities.

In high school, Louis often went to the school library to read English magazines or poems and tried to read and write down the words he saw alongside the roads. In college, Louis was disappointed to find that the class environment provided him few opportunities to learn English. Due to the lack of classroom learning conditions, Louis actively sought out foreigners to practice speaking English with at school. Louis had weekly meetings with an exchange student in a tea house where they discussed various topics, such as good restaurants:

*We made a deal like a business. He taught me English, and I taught him dialect. Whenever I meet foreigners around me, I cannot wait to talk to them because this is one of my curiosities and ways to improve my English. (Interview 1, 28 Nov. 2020*)

After graduating from university and receiving 1 month of training at L Normal University, Louis became an English teacher in a remote area. For the first 3 years, following other teachers there, he did not strive to improve his teaching and drank copious amounts of beer every day. With the help of his friend, he finally woke up and realized that he could not continue to be so decadent:

*I thought drinking a lot of beer was not good for my life, and my friend Josh, who taught in a primary school, made me want to learn English again. We tried to learn more about English together because we were pushed to learn by our interests. (Interview 1, 28 Nov. 2020*)

Despite his lack of professional learning experiences, a series of actions testify to his determination and tenacity in improving his English proficiency. Unsatisfied with his English writing, Louis tried to improve it with the help of a student who was studying at Columbia University, whom he paid and communicated with over the Internet. In addition to learning from others, Louis also purchased some online courses and spent a great deal of time reading different kinds of books. The majority of books he read were in English, and Louis mentioned that, if possible, he would like to translate them into his native language and then publish them. The considerable amount of time and monetary investment manifested in the enactment of agency in Louis’ attempts to improve his language proficiency.

At Louis’ school, the singular emphasis on test scores narrowed Louis’ space for living his “stories to live by” (Connelly and Clandinin, 1999, p. 4). The new teaching method he facilitated for the good of students’ speaking skills was considered impractical. For Louis, the school community seemed not to be a safe and open space for sharing experience but an isolated mire for him:

*The learning community is essential for us teachers. However, the teachers here are all very lazy. They do not want to talk much about how to teach students better. They even resist it. Therefore, they do not share any information. (Interview 2, 7 July 2021*)

Owing to his poor work environment, Louis became dedicated to building a WeChat online learning community consisting of voluntary teachers from different schools. Among them were five male teachers who learned English on their own in their spare time and a female teacher who had received professional and systematic English learning. In this virtual space, they communicated thoughts on what they had learned recently:

*My English was not very good, but I am good at motivating other people because this is my way to improve my English. In this group, we spoke English, shared videos, and made videos by ourselves. We had discussions once a week. Sometimes we also met face to face to talk. (Interview 1, 28 Nov. 2020*)

Nevertheless, as time went by, some problems surfaced in this WeChat learning community. It was found that members in the group rarely talked in English but enjoyed speaking in their native language. In the second interview, Louis reflected:

*Before, we were very excited to talk in English, but we do not have that passion now and we have become lazy. We are all at the same level in language skills, so we talk about what we have talked about before. We do not know how we can further improve our English. We do not make our learning environment better. (Interview 2, 7 July 2021*)

Louis’ accounts reveal that while he enacted agency in continuous learning, his intention of building an online learning community was circumscribed by the contextual constraints. Living in a town far from the city made it difficult for Louis to find an ideal “peer” to learn from. Surprisingly, although disappointed at failing to find suitable learning partners, Louis was invariably on the way to initiating a learning community for more peers:

*Many teachers in this area did not know much about online communication, online lessons, or online meetings. The schools in town bought online courses for primary school teachers. However, they had no patience for that. So right now, I am trying to facilitate the primary school teachers to learn online. (Interview 2, 7 July 2021*)

With financial support from the national education policy, Louis initiated an online English learning program for 30 teachers to learn in a community. However, it should be noted that the absence of teacher development resources nonetheless offers freedom for individuals to engage in self-directed learning, in which teacher agency plays a key role (Ollerhead, 2010). His choice of motivating more teachers to improve their language proficiency was to some extent influenced by his concerns about students’ future development and his desire to promote educational reform in Western China:

*I hope my personal experiences can influence other people. Other teachers also did not have professional learning experiences and lacked an English learning environment. I believe that other teachers can learn like me. I want to let other teachers join me, let them learn first, and then teach their students what they have learned. (Interview 2, 7 July 2021*)

Despite the challenges, it was gratifying that some learning progress among those teachers could be witnessed. The above-mentioned forms of continuous learning can overcome the contextual constraints of limited resources and lacking learning communities. Regarding this issue, it can be found that Louis’ past learning experiences played a crucial role in shaping his ideas and actions. In addition, his practice of initiating various learning communities reflects his practical evaluation of a suitable way to improve teachers’ professional development. This further echoes the finding that problematic situations can facilitate social actors’ agency (Emirbayer and Mische, 1998).

### The sources of the EFL teacher’ s agency

This section addresses the second research question on the sources of the EFL teacher’s agency. Table 2 presents the overall coding results.

**TABLE 2:** Overall coding results on the sources of agency.

Sources		Codes
Practical-evaluative sources (48)	Structural (19)	Community pressure (10), community support (9)
	Material (16)	Lack of appropriate teaching materials (8), insufficient professional development environment (8)
	Cultural (13)	Dissatisfaction with traditional teaching beliefs (7), low status of English in this area (6)
Iterational sources (32)	Life histories (19)	Louises (19)sourcesatus of English in this area (6)7), low status of English in this area (6) (8)aency in classroom instruction in
	Professional histories (13)	Inspiration from teacher training (7), student progress (6)
Projective sources (29)	Long-term goals (21)	Striving for studentsher training (7), student progress (6)status of English in this area (6) (8)aency in classroom instruction
	Short-term goals (8)	Balance between scores and language competence (4), more student engagement (4)

As shown above, the practical-evaluative dimension (48 times in total) played a fundamental role in the sources of the EFL teacher’s agency, with structural sources (19 times), material sources (16 times), and relatively fewer cultural sources (13 times). The iterational dimension (32 times in total) concerning life histories sources (19 times) and professional history sources (13 times) also exerted great influence on the sources of the EFL teacher’s agency. The projective dimension (29 times in total) played a key role as well, with long-term goal sources (21 times) being more predominant than short-term goal sources (8 times). A detailed report of these sources will be presented as follows.

#### Practical-evaluative sources

The practical-evaluative dimension was identified as a significant influence on agency, powerfully shaping or distorting decision-making and action, thus offering the possibility of either enhancing agency or inhibiting it (Priestley et al., 2015). The findings indicate that the structural, material, and cultural sources in this dimension played a vital role in the sources of Louis’ agency.

##### Structural sources: Community pressure and support

Structural sources concern community pressure and support, both of which are salient themes in this dimension (10 times each). In this study, community pressure from the schoolmaster, colleagues, and Louis’ family were among the sources of his agency.

Louis held the firm belief that education should not be narrowly focused on exams. Based on this, he proposed adopting the Phonics Teaching Method, which aims to improve students’ speaking skills. Undoubtedly, his proposal was questioned in the process of being put into practice. Faced with the schoolmaster’ s opposition, Louis insisted that “I will only do it my way” (Interview 1, 28 Nov. 2020). Nevertheless, he expressed his concern about testing requirements, stating that “the school evaluates teachers’ work only by students’ test scores. The students need to graduate” (Interview 1, 28 Nov. 2020). In this situation, while sticking to his ideas, Louis constantly adjusted his teaching practice to balance students’ test scores and their language ability. He intended to prove his choice with actions, and he believed that “It just takes time” (Interview 1, 28 Nov. 2020).

Besides this obstacle, his colleagues’ lack of understanding and support substantially impacted his work. The other teachers in Louis’ school described him as “a crazy man” and refused to teach phonics because they had never acquired that kind of teaching skills and experience. Consequently, Louis decided to train those teachers for 1 month. It seems that the lack of a positive work environment and good peer relationships, in turn, promoted Louis’ agency.

Apart from workplace stress, pressure from his wife, who achieved better teaching results working at the same school, emerged as another agency source. On the one hand, his wife questioned his devoting so much effort into teaching students speaking without seeing significant results. On the other hand, she blamed him for not teaching their son. For fear that others would question his English proficiency because of his son’s poor English grades, Louis decided to teach his son at his school and stated that he taught all the students in the kindergarten to speak English, which allowed his son to be actively engaged.

In addition to pressure from the community, some support from the community offers possible explanations for Louis’ agency. The data suggest that Louis’ achievement of agency draws to a large degree upon community support from his friends—members of the WeChat online learning community. For instance, during the first 3 years of Louis’ teaching career, it was his friend Josh who pulled him out of his predicament and provided him with unconditional support in his later teaching career.

Moreover, as Chaaban et al. (2021) pointed out, support from school leaders is an essential factor that influences teachers’ agency. In the present study, the community support from the schoolmaster at the very beginning also played a key role. When Louis put forward his proposal to attempt to teach phonics in his school, the schoolmaster agreed and allowed him to prepare related teaching material. Furthermore, the schoolmaster himself provided funds to support the promotion of the teaching material so that every student in the school could access the requisite material.

Similarly, the data of this study suggest that Louis’ belief in maximizing students’ potential and action to facilitate more teachers to learn English were derived from the support of the education policy whereby “the schools in our town bought online lessons to support all the primary school teachers” (Interview 2, 7 July 2021). Thus, it was found that support from friends and, to some extent, the support from the schoolmaster and the education policy greatly contributed to the teachers’ agency.

##### Material sources: Lack of appropriate teaching materials and insufficiency of the professional development environment

As the second typical agency source, material sources in this study refer to the lack of appropriate teaching materials (8 times) and deficiencies of the professional development environment (8 times). The findings indicate that when Louis came up with the idea of trying to teach phonics in his school, he was unable to find appropriate teaching materials. In this circumstance, Louis was forced to keep searching for available materials and was pushed to write a suitable textbook himself:

*I have already found some materials about phonics teaching on some websites. I need to make it simple and fit our students. I wanted to write a suitable book for students to learn after they graduate from primary school and before they enter junior high school. I want to make a bridge. (Interview 2, 7 July 2021*)

Likewise, when practicing some activities in class, he commented that the textbook input and resources were insufficient to engage the students and achieve the teaching goals. To address this issue, he exploited external resources and prepared extra teaching materials to engage more students:

*Students’ English proficiency levels are pretty different, so I need to prepare many materials. I also collect and want to incorporate English songs, movies, plays, and simulated situations for students. However, our students have difficulty doing the required learning activities. I think this problem is due to our English education system, which is the result of an English test system which stifles students’ imagination. (Interview 2, 7 July 2021*)

From the above quotations, it can be inferred that, in line with Emirbayer and Mische’s (1998) statement that teacher agency is triggered when faced with contradictory or problematic situations, Louis’ agentic actions were triggered by a problematic situation: a lack of appropriate teaching materials and his evaluation of students’ English proficiency levels and learning needs.

Similarly, unlike other places where available resources are abundant, the unique environment in Western China restricts teachers’ professional development. As Louis pointed out, “Few people can understand or speak English here, so it is easy for us to forget English” (Interview 1, 28 Nov. 2020). Moreover, having majored in Tourism Management at T University, Louis lacked experience in the professional and systematic learning of English, which inevitably led him to encounter some problems in his later teaching practice. Louis explained that:

*My past experiences lacked theoretical learning. I taught students just based on my personal experience. Without support from theories, I could not judge whether my way of teaching was correct or not. I did not get any professional training about how to be a good English teacher, so sometimes I felt I lost myself. (Interview 2, 7 July 2021*)

To deal with this professional dilemma, Louis engaged in extensive reading in his spare time. As he noted, repeatedly reading these books inspired him with new ideas about teaching. Additionally, Louis worked with his friend Josh to build an online WeChat learning community in an attempt to compensate for the lack of a professional development environment and maintain his passion for English teaching and learning. The second interview further revealed that, considering that many English teachers are generally exposed to an insufficient teacher professional development environment, Louis initiated one online learning program for both primary and secondary school teachers.

In summary, due to the insufficient teacher development environment, Louis’ agency of self-learning and initiating learning communities stemmed from his practical evaluation of an effective way to improve himself as an English teacher and help more teachers make progress in their classrooms.

##### Cultural sources: Dissatisfaction with traditional teaching beliefs and the low status of English in this area

Cultural sources of agency in this study concern the dominant societal beliefs and the teacher’s way of thinking, which encompass both the teacher’s dissatisfaction with traditional teaching beliefs (7 times) and recognition of the low status of English in this area (6 times). Interview data indicate that EFL teachers spent most of their time helping students prepare for tests, during which grammar teaching is strongly emphasized and students are required to engage in rote learning and recitation. Louis argued that traditional exams are not conducive to students’ future development. First, they fail to test students’ fundamental language proficiency, and the test structure hinders the cultivation of students’ listening and speaking skills. Second, these tests erase students’ creativity and imagination. Louis accordingly reflected that teachers should “teach them more for their future, get close to the students, and take note of their emotions” (Interview 1, 28 Nov. 2020).

Dissatisfied with this teaching mode, Louis kept searching for ways to make the most of students’ learning potential and interest until he discovered the Phonics Teaching Method. He described this method as an effective approach to improving students’ speaking skills. However, Louis’ intention to practice this method at school seemed counter to the school’s goal of improving students’ test scores. Therefore, despite Louis’ efforts to promote the Phonics Teaching Method, the other teachers did not go along with teaching phonics. In this situation, Louis was on the way to proving that teaching phonics is a sensible choice to help students learn English, and he firmly believed that students would be benefited in the future:

*I need time. However, the parents and other teachers do not want to spend time on this endeavor. They just want to see a good result in a short period. They never see how the students will be unconsciously influenced in the future. (Interview 1, 28 Nov. 2020*)

With this conviction, Louis established an English learning corner to teach students phonics on weekends and guided them to learn about artificial intelligence to broaden their horizons. This finding is consistent with the view that agency is shaped and reshaped by various sociocultural factors such as teaching contexts, teachers’ beliefs about teaching, and their expectations of students (Le et al., 2020).

Additionally, the interview data indicate that the examination policy greatly influenced the beliefs and practices of this EFL teacher. In 2016, the local government reformed the junior high school entrance examination, for which the scores for English as a subject dropped significantly. Under the influence of the examination policy, there is insufficient cohesion between English learning and teaching in primary and junior high school, and seventh-grade students are not well-adapted to English learning. Much worse, since little attention has been placed on English, many primary school English teachers are required to teach another subject like Chinese or science, which further reduces their enthusiasm for English teaching. Louis recalled one teacher training experience with his colleagues:

*Most of the teachers participated in the teacher training just for fun because they felt there was no need to teach the students English. According to them, children know little about English and never want to learn, so students consider English to be useless. Based on this idea, it seems that some teachers have already given up on teaching English. (Interview 1, 28 Nov. 2020*)

Accordingly, Louis expressed his determination to help these primary school teachers regain their enthusiasm for teaching by initiating learning communities. Based on his assertation that learning problems arise from the education policy, Louis said that his plan for the next 5 years is to write a book that serves as a bridge to create a cohesive policy between primary and junior high school. In addition, he revealed his desire for his leadership to be reflected when addressing the existing issues in education policy: “I hope the government can figure out what kind of English teaching environment we should have and understand that the examination system should be reformed” (Interview 2, 7 July 2021). In conclusion, it is clear that because of the low status of English in this area due to the influence of the education policy, Louis’ agency of improving the status of the English subject sprouted and grew.

#### Iterational sources

With respect to the iterational dimension, the data analysis indicates that life histories consist of the teacher’s personality, authentic communication with native speakers, and disappointment with high school learning experiences. His professional histories comprise inspiration from teacher training and students’ progress.

##### Life history sources: Louis’ personality, authentic communication with native speakers, and disappointment with the high school learning environment

As regards the more general life history of this teacher, his personality (13 times) is deemed more significant than authentic communication with native speakers (6 times) and disappointment with high school learning environment (2 times) in shaping his teacher agency. Agency with regard to personality refers to the stable inclination to make choices and engage in actions to control one’s life or environment (Goller and Harteis, 2017).

Data analysis reveals that Louis’ personality, i.e., being inquisitive, adventurous, innovative, persistent, and ambitious, was the most salient source of his agency with regard to life history. In the two interviews, it was not difficult to find that Louis was an inquisitive teacher, which further explains his agentic actions of continuous investment in autonomous learning. Apart from extensive reading, Louis’ inquisitiveness was also reflected in his willingness and initiative to learn from others. He explained that it was all because of his inner passion, something that he felt came naturally to him:

*I would like to communicate with people and exchange ideas. I only want to focus on what interests me. When I came to S City this time, I met a child whose English was excellent, so I was curious about her. Then I went to communicate with her mother. I like this kind of thing. We can learn, not only from professors but also learn from small children. (Interview 1, 28 Nov 2020*)

Additionally, his adventurous and innovative spirit was reflected in applying what he had learned, seen, and felt to his teaching practice. Whether it was the novel Phonics Teaching Method or the idea of creating an English learning environment, he was the first teacher to put it into practice. As he describes in his own words, “We need to step out of our comfort zone. It was very important” (Interview 1, 28 Nov. 2020). Furthermore, his innovative personality spurred him to initiate learning communities and practice various classroom learning activities. When Louis’ actions were constrained by the physical environment and hindered by his colleagues, it was his perseverance that worked as his spiritual support. Additionally, in aspiring to become a professor at T University, Louis admitted that he was ambitious. It appears that it was precisely such an agentic personality that caused Louis to engage in agentic actions.

Moreover, interview data indicate that Louis’ past communication experiences with native speakers were an indispensable source of agency. Evidence suggests that it was his opportunities to communicate with native foreigners that developed his desire to learn English. When entering university, Louis constantly sought out exchange students to improve his spoken English through language practice. Surprisingly, Louis found the charm of learning English, exclaiming “English gives a person power!” (Interview 1, 28 Nov. 2020). This kind of emotional experience inspired him to improve his spoken English and later become devoted to improving students’ speaking skills.

As Sisson (2016) noted, teachers’ negative learning experiences in their school life are of paramount importance to their present teaching practices. The interview data showed that Louis’ teaching agency was also directly affected by his English learning experiences. When asked why he paid so much attention to students’ speaking skills, Louis replied:

*Many teachers said that they could not speak English and their English pronunciation was not good. It was because nobody taught them English when they were students. Neither did I. Nobody taught me. There was one English teacher who always taught grammar every day. Also, I never heard any English words outside the textbook. The textbook was outdated, and teachers only taught those words in it. (Interview 1, 28 Nov. 2020*)

As his explanation shows, it was these negative English learning experiences during his time in school that reminded him why it was important to become a responsible English teacher and prompted him to exert his teaching agency. Louis’ past learning experiences also influenced his teaching attitude. In his mind, tests were not good for students’ future but instead served as an obstacle to language learning. Considering that students could hardly have a bright future solely relying on textbook content, Louis aspired to create a learning environment and provide students with sufficient support.

##### Professional histories sources: Inspiration from teacher training and students’ progress

Professional histories refer to teachers’ education and the accumulated experience of being a teacher (Priestley et al., 2015). In this study, this teacher’s professional histories include his inspirations derived from teacher training (7 times) and his students’ progress (6 times).

When moving to the city in 2012, Louis met his friend Josh, who had just returned from TIP teacher training. Thanks to Josh, he became acquainted with the Phonics Teaching Method. In 2014, Louis himself participated in this program in B City. Since then, Louis has searched for related teaching materials for phonics and created an “English only” teaching environment. Moreover, after returning from training in S City, he incorporated various learning activities in class. In his words, this training experience enabled him to see a larger world and strengthened his determination to improve students’ spoken English.

Apart from personal training experiences, students’ performance also matters with regard to Louis’ agency. Although the work environment constrained Louis’ ability to teach the Phonics Teaching Method, he addressed this challenge by seeking hope from his students’ progress. Since students were always encouraged to speak English in class, Louis expressed his delight when witnessing their progress in speaking:

*Two years ago, I met one of my former students. He had graduated from high school, and his English was excellent. While in middle school, he did not like reciting but liked telling stories in his native language. So in my class, I let him tell some stories in English. At that time, I found that he could talk with me in English, whereas he could not before, so I believe this method will work for other students in the future. (Interview 2, 7 July 2021*)

As can be seen, the progress of students’ speaking gave Louis the confidence to elicit changes in other students and further strengthened his perseverance in pushing his students to speak English. It seems that even if only one student can speak English like him in the future, his enthusiasm for teaching will not fade. Later, to balance the students’ test scores and their learning ability, he made adjustments and improvements to his teaching approach. His decisions were related to the repertoire of outcomes from his prior teaching practices. This echoes Le’s (2020) study, which revealed that students’ outcomes contributed to the teachers’ employing mandated activities.

#### Projective sources

The projective dimension of teacher agency concerns students’ aspirations regarding their work, which involves both long- and short-term goals. The findings show that sources from long-term goals (21 times in total) are much more frequent than short-term goals (8 times in total). Among the long-term goals, striving for students’ future development (14 times) is a vital motivation. This section investigates how these different goals contributed to the teacher’s agency.

##### Long-term goal sources: Striving for students’ future development, pursuing personal professional development, and promoting educational reform

Long-term goals are commonly identified as aspirations rooted firmly in teachers’ values and beliefs (Priestley et al., 2015). In this study, these involve striving for students’ futures (14 times), pursuing personal professional development (5 times), and promoting educational reform (2 times). Lasky (2005) noted that teachers’ aspirations might be entirely positive, relating to the development and welfare of students and leading to the agency that projects students’ interests. In this study, Louis continuously mentioned the phrase “students’ future development,” which evidenced that his aspirations were inseparable from his students. Unlike other teachers, who exclusively focused on whether students were obedient or whether they earned high test scores, Louis cared more about students’ futures and how to maximize their potential. This aspiration empowered him to teach students knowledge about artificial intelligence, which aimed to broaden students’ horizons. Moreover, unsatisfied with just teaching to tests and prioritizing core competencies, Louis experimented with various ways of improving students’ overall language skills.

Besides striving for students’ future development, the teacher’s long-term goals included personal professional development. Among his past experiences, his passion for learning English emerged as the most impressive feat. When asked why he did everything he could to improve his English, he responded:

*I am ambitious. I planned to pursue a higher degree. I want to be a professor at T University someday. How can I become a university teacher? I do not have any research experience. I have to find my way to do research. Now, I am only focused on finding my English teaching model. (Interview 1, 28 Nov. 2020*)

Clearly, he constantly explored autonomous learning and creative teaching for his professional development that. Based on the phonics book he had written for his students, Louis expressed his future goal of writing a better version.

Moreover, Louis decided to try new teaching methods and incorporate various learning activities after realizing that the teaching and examination modes were not conducive to improving students’ English language proficiency. Even worse, the reform of the junior high school entrance examination lowered the status of English as a subject, diminishing schools, teachers, and students’ enthusiasm for teaching and learning. In this situation, Louis firmly asserted the need for urgent reform of the examination system.

It appears that different long-term goals are projected onto the current teaching practice, which prompts the teacher to perform different agentic behaviors. Louis’s projections concerning his teaching goals appear to have their roots in his iterational experiences, namely, his prior learning and teacher training experiences. However, these were circumscribed by the context in which he worked. This reflects that in a temporal sense, the projective aspect of the agency was shaped by the iterative aspect but constrained by the practical-evaluative aspect (Priestley et al., 2015).

##### Short-term goal sources: Achieving a balance between scores and language competence and increasing students’ engagement

Priestley et al. (2015) indicated that short-term goals are more narrowly instrumental, and Louis’ teacher agency was shaped by short-term aspirations to achieve a balance between scores and language competence (4 times) and to engage more students (4 times). In the second interview, Louis confessed that he was no longer teaching phonics because it resulted in unsatisfactory student test scores. He further stated that he had managed to create a balance between students’ scores and language competence:

*I care more about their tests now. The students themselves need to graduate. Before, I only cared about their language skills and let them talk and play. Now, I am thinking about what kind of test they must take, so we should learn about that. (Interview 2, 7 July 2021*)

Surprisingly, the teaching adjustment indeed improved the mid-term test scores of the students in Louis’ class. In this regard, Louis revealed his delight with students’ progress. It can be seen that Louis’ short-term goal works as a critical projective source of his agency, that is, flexibly adapting the teaching focus to ensure students successfully graduate.

Moreover, Louis wanted to improve students’ learning fulfillment and satisfaction by increasing student engagement in class. Practicing a completely different approach to teaching than other teachers, Louis admitted that he was satisfied with his teaching environment. However, it was also because his teaching style differed from other teachers that Louis faced some thorny problems. For instance, some students found it hard to catch up with him, so they lost their passion for learning. Other students even ran out of the classroom, disrupting the classroom discipline. Nevertheless, Louis said he would continue teaching the class as long as one student listened to him. His agency also manifested in his desire to boost student engagement in the class.

*I want to focus on all the students, but there are more than 40 students in a class, so I divided the class into different groups and assigned them different tasks—some exercises were easier, such as writing sentences. Then I found some of them knew something. Some boys were very clever, but they do not have a passion for learning. (Interview 2, 7 July 2021*)

Therefore, to attract the attention of these students and involve them in class, Louis tried to incorporate initial teaching activities. Imitating the dialogue in short videos and reading lines from Shakespeare’s plays were the most popular activities for students. It can be inferred that Louis’ desire to engage more students in classroom activities serves as a crucial source of his agency that functions like a sponge, constantly absorbing new ideas into his teaching. Therefore, this study reveals that the teacher’s short-term goal sources of agency primarily resulted from his practices in the practical-evaluative aspect.

## Discussion

### Manifestation of the EFL teacher’s agency

The two agency stories depict how this EFL teacher Louis enacted his agency in his passionate exploration of adaptive teaching and continuous investment in autonomous learning, particularly with respect to the different approaches Louis took to actively exert his agency in such an under-resourced environment in Western China. A close look at the stories reveals that although this EFL teacher’s high level of the agency was manifested throughout his teaching, it was affected by some restrictive factors. Specifically, teacher agency in the two stories has in common the fact that, in the process of enacting agency, it was easy for the EFL teacher to fall into a state of restricted agency (Wei and Chen, 2019). This finding also aligns with Biesta et al.’s (2017) statement that teachers can experience agency and powerlessness simultaneously.

For instance, Louis was pressured to teach to the test rather than actively improving students’ language skills. Meanwhile, his goals for practicing newly learned learning activities in class failed to some extent due to students’ low language proficiency. This finding indicates that teachers’ agency is restricted when they subjectively identify with certain advanced perspectives and psychologically show a strong motivation for change but are limited by certain personal or external factors that limit or lower their agency. Surprisingly, in this study, Louis’s inaction was not identified when his agency was “restricted,” whereas Wei and Chen (2019) found that the contradiction between “perspective and action” lowered the level of agency and manifested itself in reality as “negative coping.” In their study, participating teachers with strong agency in professional development had encountered a sense of frustration and powerlessness in their teaching practice, thus finally falling into a helpless state of “restricted agency.”

For Louis, however, the data show that he always addressed problematic situations by turning to or devising another method to continue enacting his agency. For example, he paid more attention to students’ test scores by assigning some writing tasks in consideration of the practical needs of graduation. Similarly, when reflecting on students’ low language proficiency, Louis organized an online learning community to facilitate over 30 teachers’ English learning. These actions further confirm that Louis insistently enacted his high levels of agency to solve problems in an under-resourced environment.

Additionally, high levels of agency were manifested in this teacher’s perspectives and actions of resistance to be changed. As revealed in this study, Louis’ stubbornness manifested in his insistence on teaching extra knowledge to students, despite the pressure of the mandate to “teach to the test.” This finding aligns with the claim that resistance is considered a form of agency that can be modified and transformed into constructive forms of agency (Gonzales, 2014). In addition, this finding echoes Buchanan’s (2015) finding that “stepping up” reflects teachers’ agency of trying out new ideas and taking up additional roles, while “pushing back” involves teachers’ agency of rejecting or re-configuring practices and policies with which they do not agree. Unlike other teachers, who were solely concerned about students’ grades, Louis reconsidered a “safe way” to balance students’ scores and language competence. This kind of agency could be the reason for the teacher’s dissatisfaction with the education policy and disagreement with the teaching beliefs of his colleagues and superiors. Therefore, under this circumstance, the EFL teacher’s agency was primarily manifested in his resistance to the policy-influenced teaching perspectives and actions of schools and other teachers.

Furthermore, it should be noted that teacher agency can be observed in aspects like learning, teaching, and research. Compared to the relative consistency of his engagement with teaching and learning, the EFL teacher showed “restricted agency” in research, which is more closely linked to his contextual constraints. Working within an unfavorable situation, Louis confessed that his willingness to conduct research was impeded by a lack of knowledge of related learning theories. His response reflects a general lack of agency in conducting research for professional development, particularly when limited resources are available (Tao and Gao, 2017).

### Complex sources of the EFL teacher’s agency

In general, echoing Emirbayer and Mische’s (1998) statement that human agency is triggered when faced with problematic situations, this study shows that a problematic situation in this under-resourced environment triggered this teacher’s modification of the teaching method. Most of the students in the class could not understand his teaching and performed poorly on tests. It is also worth noting that Louis’ agency was triggered by the problematic situation he worked in—an environment without sufficient teaching materials and advanced teaching equipment. In the present study, it was precisely the existence of these problems that stimulated Louis’ agency to compile appropriate teaching materials for the students, thus enabling them to learn more about advanced technology in the form of videos. This finding is inconsistent with Chaaban et al.’s (2021) claim that a lack of technological devices, Internet connectivity, and stable electricity are significant factors hindering teachers from taking action toward students learning. However, it completely confirms the claim that teachers feel more creative in problematic situations, thus achieving higher levels of agency (Priestley et al., 2015), as teachers in under-resourced environment are provided with more possibilities to enact agency.

Furthermore, interview data revealed that the gap between the EFL teacher’s past experiences and his goals served as an intrinsic motivation to arouse his agency while making certain changes, which echoes Liu’s (2020) conclusion that teachers’ agentic actions originate from the gap between the iterational and the projective dimensions. Similarly, as Pei and Yang (2019) pointed out, teachers’ learning experiences could lead their teaching focus to shift from a test orientation to an emphasis on students’ learning and well-being. In this study, Louis’ learning experiences in high school and teacher training impacted his beliefs on students’ future development. Therefore, the gap between what he had witnessed and what he would like to achieve triggered Louis’ autonomous learning and creative teaching agency. It is his long-term goal and passion for changing students’ future that drives his development of teacher agency.

Additionally, the data provide ample evidence that the EFL teacher’s interaction with the environment he worked in serves as the primary source of his agency. Four-fifths of the sources were derived from restricted situations. Despite the imposed constraints, Louis willingly stepped out of his comfort zone, experimenting with his teaching with both dedication and passion (Le et al., 2020). It confirms the interplay of person and environment that shapes teacher agency (Priestley et al., 2015). In addition, it further reveals that it is this teacher’s belief and identity that help trigger his agency in this under-resourced environment where more resources are not available.

## Conclusion

The current study explored how an EFL teacher enacted his agency in teaching and learning and identified the sources of his agency in an under-resourced teaching environment in Western China. This narrative study showed that Louis’ agency was manifested in two aspects—namely, passionate exploration of adaptive teaching and continuous investment in autonomous learning. It further revealed that his agency was mainly derived from three dimensions: practical-evaluation, iteration, and projection. Through the lens of narrative inquiry, a vivid picture was drawn of how an EFL teacher’s agency was mutually formed by various sources in his particular institutional context. The findings derived from one EFL teacher are eminently situated in this under-resourced teaching environment, which cannot be generalized to other contexts. However, this study assumes a qualitative approach, for which description, understanding, and interpretation of communication are the primary goals (Bloomberg and Volpe, 2018). The findings of this study are significant for inspiring more researchers to focus on teacher agency in similar contexts.

The study has the following implications. First, it is suggested that EFL teachers in under-resourced teaching environments take the initiative to seize opportunities to learn and participate in teacher training, thereby enriching their knowledge and experiences. In addition, EFL teachers in these contexts should establish their own long term professional development goals and timely update and absorb advanced teaching beliefs and methods so as to have more agency in their career. Second, it is advised that school administrators in under-resourced teaching environments create a culture of respect for professionalism in schools, empower teachers with autonomy in their work, and transform teachers from passive performers to active professional practitioners. Moreover, professional development support for teachers and the building of teacher communities are needed. More importantly, there is an urgent need for educational policymakers to reexamine the issue of English education and create a more reasonable and effective examination system that contributes to promoting students’ English learning.

This study has several limitations. Since this study is largely based on interviews, it is unlikely to capture the developmental process of the teacher’s agency formation. Additionally, the retrospective focus of the interviews may affect the accuracy of the interviewees’ responses due to time lag and only provides constructed data rather than what precisely happened (Tao and Gao, 2017). Thus, longitudinal studies are encouraged to explore the changes in teachers’ agency through which their learning and teaching experiences developed. Future researchers can also consider involving more EFL teachers from different teaching environments to draw a broader picture of teacher agency.

## Data availability statement

The original contributions presented in this study are included in the article/supplementary material, further inquiries can be directed to the corresponding author.

## Ethics statement

The studies involving human participants were reviewed and approved by Graduate School, Soochow University. The patients/participants provided their written informed consent to participate in this study.

## Author contributions

All authors listed have made a substantial, direct, and intellectual contribution to the work, and approved it for publication.

## Footnotes

1 The original word was “myself.” Due to many grammatical errors in the participant’s English expressions, the author has modified his words in the following quotations to enhance readability.

2 The Total Immersion Program, a fully enclosed oral English training project that reflects the Great Western Development Strategy, aims to improve the oral level of primary and middle school English teachers in the western region and update their teaching beliefs, thereby comprehensively improving the quality of elementary education in the region.

3 The Phonics Teaching Method is applicable for reading and vocabulary lessons, which teaches children how to read and write and helps children hear, identify, and use different sounds that distinguish one word from another in the English language.
